# Robot-Assisted Radical Prostatectomy: Concordance and Correlation Between Preoperative Positron Emission Tomography-Computed Tomography With Prostate-Specific Membrane Antigen (PET-CT PSMA) and Final Histopathological Report

**DOI:** 10.7759/cureus.82019

**Published:** 2025-04-10

**Authors:** Jose R Vazquez Gonzalez, Carlos M Vasquez Lastra, Christian I Villeda Sandoval, Brandon Martinez Torres, Francisco A Santacruz Chavez

**Affiliations:** 1 Urology, Centro Medico ABC, Mexico City, MEX; 2 Urologic Oncology, Centro Medico ABC, Mexico City, MEX; 3 Urology, Universidad Nacional Autonoma de Mexico, Mexico City, MEX; 4 Medicine, Universidad Anahuac Mexico Norte, Mexico City, MEX

**Keywords:** concordance, correlation, da vinci, diagnostic accuracy, histopathology, pet-ct psma, prostate cancer, radical prostatectomy

## Abstract

Background: Positron emission tomography-computed tomography with prostate-specific membrane antigen (PET-CT PSMA) has revolutionized the preoperative staging of prostate cancer, particularly for assessing seminal vesicle and lymph node invasion. However, its correlation and agreement with definitive histopathology remain unclear.

Objective: The study aimed to evaluate the diagnostic performance, correlation, and agreement between PET-CT PSMA and final histopathological findings for seminal vesicle and lymph node invasion in patients undergoing robot-assisted radical prostatectomy.

Methods: This retrospective, single-center study included 194 patients who underwent PET-CT PSMA and radical prostatectomy. Sensitivity, specificity, positive predictive value (PPV), negative predictive value (NPV), and overall accuracy were calculated. Correlation (Spearman's rho) and agreement (kappa coefficient) between PET-CT PSMA and final histopathology were analyzed.

Results: Seminal vesicle invasion was detected in 11.9% of cases by histopathology and 12.4% by PET-CT PSMA, while lymph node invasion was reported in 4.8% and 10.3%, respectively. PET-CT PSMA exhibited high specificity (91.23% for seminal vesicles, 92% for lymph nodes) and high NPVs (91.76% and 96.84%, respectively), but low sensitivity (39.13% and 40%) and low PPV for lymph nodes (20%). Correlation between PET-CT PSMA and histopathology was low for seminal vesicle invasion (rho = 0.298; p = 0.0001) and lymph node invasion (rho = 0.232; p = 0.017). Agreement was also poor for seminal vesicle invasion (kappa = 0.298) and lymph node invasion (kappa = 0.217).

Conclusion: PET-CT PSMA demonstrates high specificity and NPV but low sensitivity, correlation, and agreement with final histopathology. While useful for ruling out disease, its positive findings should be interpreted with caution.

## Introduction

Prostate cancer is the second most common cancer in men and one of the leading causes of mortality worldwide. In 2020, more than 1.4 million new cases and approximately 375,000 deaths were reported, according to the World Health Organization (WHO) [1,2]. In Mexico, prostate cancer represents a growing public health concern, with an estimated 25,000 new cases and over 7,500 deaths annually, figures that continue to rise [3]. Accurate staging is essential to determine the optimal treatment strategy and improve oncological outcomes.

Positron emission tomography-computed tomography with prostate-specific membrane antigen (PET-CT PSMA) has revolutionized the staging of prostate cancer by enabling a more precise detection of metastatic disease. Its ability to identify lymph node and bone metastases has significantly enhanced surgical planning and clinical decision-making [4-7]. Compared to conventional imaging modalities, PET-CT PSMA offers higher sensitivity and specificity, making it a widely adopted tool in urological practice [6]. However, most studies evaluating its performance have primarily focused on diagnostic accuracy metrics, including sensitivity, specificity, predictive values, and overall accuracy, leaving correlation and agreement between preoperative PET-CT PSMA findings and final histopathology largely unexplored.

At our center, PET-CT PSMA is routinely performed before robot-assisted radical prostatectomy to improve the identification of seminal vesicle and lymph node invasion. However, the extent to which preoperative PET-CT PSMA findings correlate with final histopathological results remains unclear. While some studies have reported high specificity for detecting metastases, the actual concordance between PET-CT PSMA and postoperative histopathology has yet to be fully established [8-12].

Therefore, the objective of this study is to assess the diagnostic accuracy, correlation, and agreement between preoperative PET-CT PSMA findings and final histopathological results in patients undergoing robot-assisted radical prostatectomy, with a specific focus on seminal vesicle and lymph node invasion.

## Materials and methods

Study design, study period, and selection criteria

This is a retrospective, single-center study based on data collection from medical records. We included records of men over 45 years old with a confirmed diagnosis of prostate cancer who underwent disease staging with gallium-68 (68Ga)- and fluorine-18 (18F)-PSMA PET-CT and subsequently underwent robot-assisted laparoscopic radical prostatectomy with the Da Vinci system between March 2022 and June 2024 at Centro Medico ABC in Mexico City.

We included all patients regardless of their Gleason grade, International Society of Urological Pathology (ISUP) group, or National Comprehensive Cancer Network (NCCN) risk classification. We excluded patients with synchronous neoplasms or histologies other than acinar prostate adenocarcinoma. Clinical records lacking the necessary information for the study, such as histological results or 68Ga- and 18F-PSMA PET-CT findings, were also excluded.

Histopathological and 18F-PSMA/68Ga-PSMA PET-CT definitions

All histological definitions followed the NCCN Clinical Practice Guidelines in Oncology recommendations [13]. The Gleason classification was collected and reported by score and risk group, categorizing patients into low/very low risk (Gleason score ≤ 6), favorable intermediate (Gleason score 7 (3 + 4)), unfavorable intermediate (Gleason score 7 (4 + 3)), high risk (Gleason score 8), and very high risk (Gleason score 9-10).

Similarly, all definitions for 18F-PSMA/68Ga-PSMA, such as lesion intensity, number of lesions, and normal vs. abnormal uptake patterns, were consistent with most published studies [8,9,14-17]. We included three definitions of PET-CT positivity for neoplasia: maximum standardized uptake value (SUVmax) >2.5 [14], tracer uptake higher than the background [8,15-17], and tracer uptake higher than the liver uptake [9].

Statistical analysis

The Kolmogorov-Smirnov test was used to assess the normality of variables. Since numerical variables did not follow a normal distribution, they were reported as medians and ranges, while categorical variables were reported as frequencies and percentages.

The primary outcome was to analyze the correlation and agreement between the final histopathological findings and PET-CT PSMA results, specifically in terms of seminal vesicle invasion and lymph node invasion. Spearman's correlations and kappa agreement were used. Correlations were classified as weak (0.00-0.29), moderate (0.30-0.59), and strong (≥0.60), while kappa agreement was classified as poor (<0.20), fair (0.21-0.40), moderate (0.41-0.60), substantial (0.61-0.80), and almost perfect (>0.80).

All statistical tests were bivariate, and a p-value of <0.05 was considered significant. Missing values were allowed in histopathology results for one structure (seminal vesicles or lymph nodes), and a complete case analysis approach was applied.

All analyses were performed using RStudio Version 2023.12.1+402 (Posit Software, Boston, MA, USA), and specific R packages (R Foundation for Statistical Computing, Vienna, Austria) such as ggplot were used for graph generation.

Ethical considerations

This study complies with the ethical principles established in the Declaration of Helsinki, which sets guidelines for ethical research in human subjects. Additionally, it adheres to the regulations established by the General Health Law on Human Research in Mexico. Since this is a retrospective study, the Ethics Committee of the Directorate of Education and Health Research granted an exemption for informed consent.

## Results

Population characteristics and intraoperative histology

A total of 194 patients who underwent PET-CT PSMA (68Ga and 18F) and robot-assisted radical prostatectomy with the Da Vinci system were included. The median age was 67 years (range: 43-82 years). The total Gleason score, based on transrectal biopsy, had a median of 7 (range: 6-9). The primary and secondary Gleason components had a median of 4 (range: 3-5) for both (Table 1).

**Table 1:** Clinicopathological characteristics of patients with PET-CT PSMA (n = 194) This table summarizes the clinicopathological characteristics of 194 patients who underwent PET-CT PSMA and radical prostatectomy assisted by the Da Vinci robotic system. PET-CT PSMA: positron emission tomography-computed tomography with prostate-specific membrane antigen; TRPB: transrectal prostate biopsy; HP: histopathological

Variable	Median (range) or n (%)
Age (years)	67 (43-82)
Total Gleason score (TRPB)	7 (6-9)
Primary Gleason component (TRPB)	4 (3-5)
Secondary Gleason component (TRPB)	4 (3-5)
Gleason score (transurethral biopsy), n (%)	
Very low/low	41 (21.1%)
Favorable intermediate	55 (28.4%)
Unfavorable intermediate	35 (18%)
High	46 (23.7%)
Very high	17 (8.8%)
Prostate-specific antigen (ng/mL)	7.22 (0.21-4357)
Partin tables, % (median, range)	
Organ-confined disease	43 (5-94)
Extraprostatic extension	33 (9-93)
Seminal vesicle invasion	7 (1-42)
Lymph node invasion	5 (0-48)
Total Gleason score (final HP)	7 (6-9)
Primary Gleason component (HP)	4 (3-5)
Secondary Gleason component (HP)	4 (3-5)
Final histopathology Gleason score, n (%)	
Very low/low	15 (7.7%)
Favorable intermediate	74 (38.1%)
Unfavorable intermediate	60 (30.9%)
High	21 (10.8%)
Very high	24 (12.4%)

Regarding risk classification based on transrectal biopsy, 21.1% of patients were classified as very low/low risk, 28.4% as favorable intermediate, and 18% as unfavorable intermediate. The high-risk and very-high-risk categories accounted for 23.7% and 8.8% of patients, respectively.

The prostate-specific antigen (PSA) level had a median of 7.22 ng/mL (range: 0.21-4357 ng/mL) (Table 1). According to Partin tables, 43% of patients had organ-confined disease, while 33% exhibited extracapsular extension, and 7% presented seminal vesicle invasion. Lymph node invasion was observed in 5% of cases.

Final histopathological report findings

The Gleason score, based on the final histopathological report, had a median of 7 (range: 6-9), with similar values for the first and second Gleason components. Regarding definitive histology, 7.7% of patients were classified as very low/low risk, 38.1% as favorable intermediate, and 30.9% as unfavorable intermediate. Patients in the high-risk and very-high-risk categories accounted for 10.8% and 12.4%, respectively (Table 1).

Of the total patients, 50.9% received treatment with bicalutamide. The median surgical time was 240 minutes (range: 120-450 minutes). Postoperative complications were absent in 97.4% of patients. Reported complications included colonic injury in 1% of patients, acute kidney injury in 0.5%, and urosepsis in 0.5%. No cases of abdominal wall collections, wound dehiscence, or ileal perforation were reported. Postoperative bleeding had a median of 250 mL (range: 50-700 mL). Surgical margins were negative in 50.9% of patients and positive in 49.1% (Table 2).

**Table 2:** Surgical and postoperative characteristics of patients with PET-CT PSMA (n = 194) This table summarizes the clinicopathological characteristics of 194 patients who underwent PET-CT PSMA and radical prostatectomy assisted by the Da Vinci robotic system. PET-CT PSMA: positron emission tomography-computed tomography with prostate-specific membrane antigen

Variable	Median (range) or n (%)
Bicalutamide use (n = 108)	55 (50.9%)
Surgical time (minutes)	240 (120-450)
Surgical margins in final histopathological report (n = 106)	
Negative	54 (50.9%)
Positive	52 (49.1%)
Prostate volume by PET (mL)	51 (20-220)
Complications, n (%)	
None	189 (97.4%)
Abdominal wall collection	0 (0%)
Wound dehiscence	0 (0%)
Colon injury	2 (1%)
Acute kidney injury	1 (0.5%)
Ileal perforation	0 (0%)
Urosepsis	1 (0.5%)
Postoperative blood loss (mL)	250 (50-700)

Observed agreement, expected agreement by chance, and diagnostic performance of PET-CT PSMA

In the final histopathological report, seminal vesicle invasion was observed in 11.9% of cases, while lymph node invasion was reported in 4.8% of patients. In PET-CT PSMA, 24 patients (12.4%) and 20 patients (10.5%) showed seminal vesicle and lymph node invasion, respectively. Extraprostatic extension by PET-CT PSMA was reported in 13 patients (6.7%) (Table 3).

**Table 3:** Histopathological findings and PET-CT PSMA results This table compares the final histopathological findings with PET-CT PSMA results in 194 patients who underwent radical prostatectomy assisted by the Da Vinci robotic system. PET-CT PSMA: positron emission tomography-computed tomography with prostate-specific membrane antigen; HPR: histopathological report

	n = 194
Invasion to seminal vesicles, HPR (n, %)	23 (11.9%)
Invasion to lymph nodes, HPR (n, %) (n = 105)	5 (4.8%)
Invasion to seminal vesicles, PET-CT PSMA	24 (12.4%)
Invasion to lymph nodes, PET-CT PSMA	20 (10.3%)
Extraprostatic extension, PET-CT PSMA	13 (6.7%)

**Figure 1:**
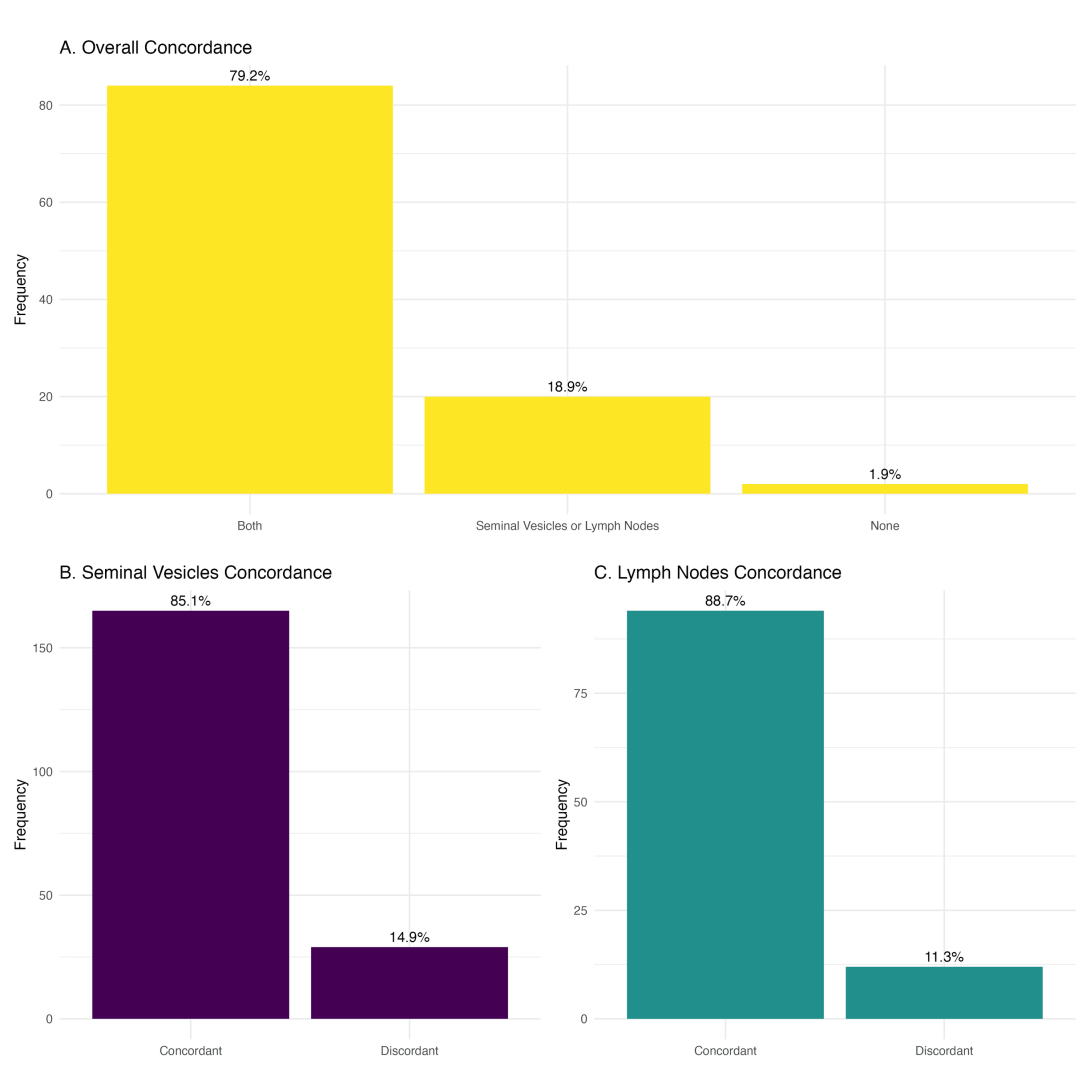
Overall and specific concordance observed between PET-CT PSMA and the histopathological report This figure illustrates the overall and specific concordance between preoperative PET-CT PSMA findings and the final histopathological report. The upper graph categorizes patients into three groups: (A) full concordance ("both"), (B) partial concordance ("seminal vesicles or lymph nodes"), and (C) no concordance ("none"). The lower graphs depict specific concordance for seminal vesicle and lymph node invasion. PET-CT PSMA: positron emission tomography-computed tomography with prostate-specific membrane antigen

The agreement between PET-CT PSMA and final histopathological report findings was 85.1% for seminal vesicle invasion and 88.7% for lymph node invasion (Figure 1). Concordance between PET-CT PSMA and final histopathology occurred in both structures (seminal vesicles and lymph nodes) in 79.2% of cases and in one or the other (seminal vesicle or lymph node concordant) in 18.9%, and in 1.9% of cases, there was no concordance in either structure (Figure 1).

**Figure 2:**
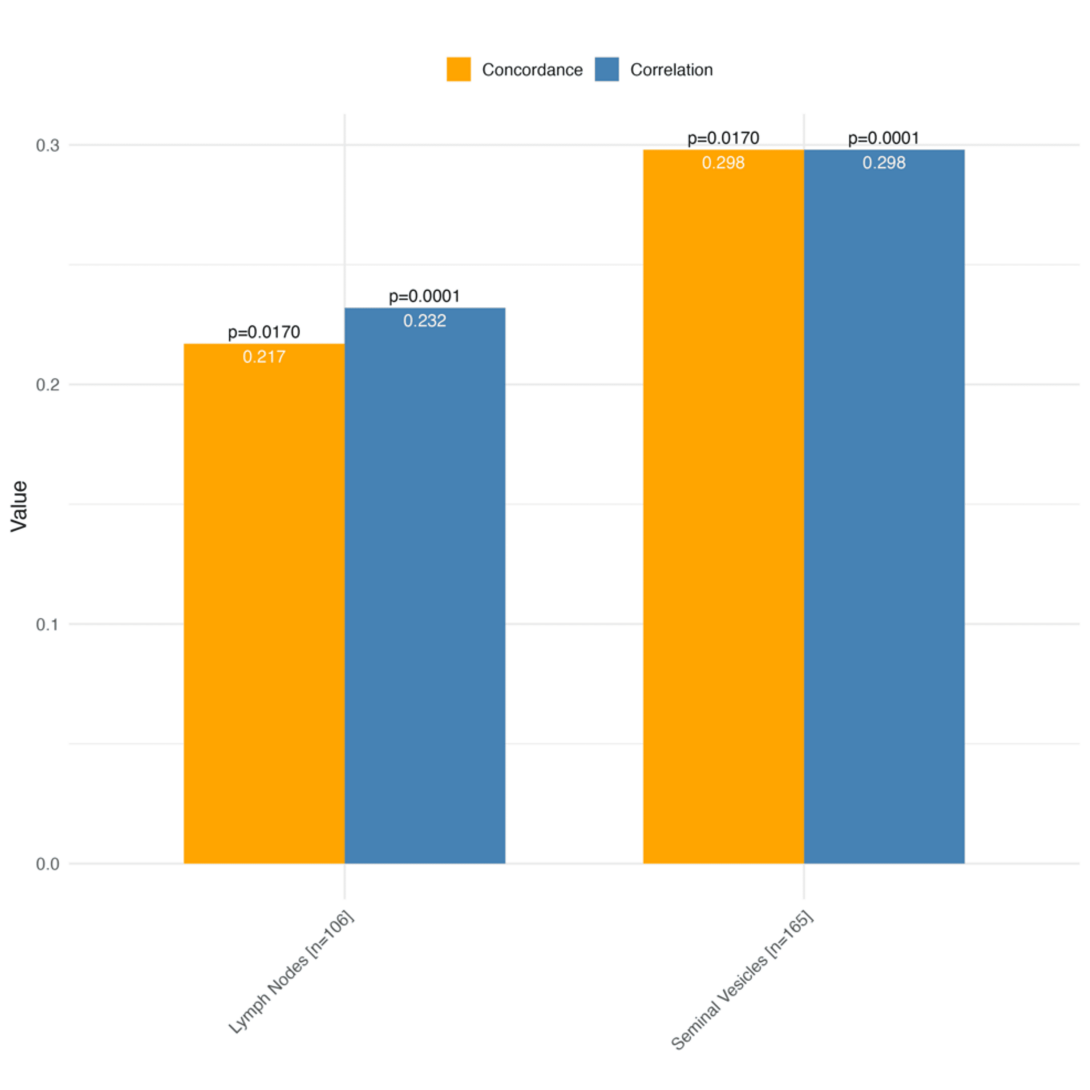
Correlation and agreement analysis between PET-CT PSMA and histopathology This figure presents the correlation (Spearman's rho) and agreement (kappa coefficient) between PET-CT PSMA findings and the histopathological report for seminal vesicle invasion and lymph node invasion. Correlation and agreement coefficients are displayed along with their respective p-values. PET-CT PSMA: positron emission tomography-computed tomography with prostate-specific membrane antigen

In the correlation and agreement analysis between PET-CT PSMA and final histopathology, the correlation for seminal vesicle invasion was low (rho = 0.298; p = 0.0001), while agreement was also low (kappa = 0.298; p = 0.017). For lymph node invasion, the correlation was 0.232 (rho = 0.298; p = 0.0001), and agreement was 0.217 (kappa = 0.298; p = 0.017) (Figure 2).

**Figure 3:**
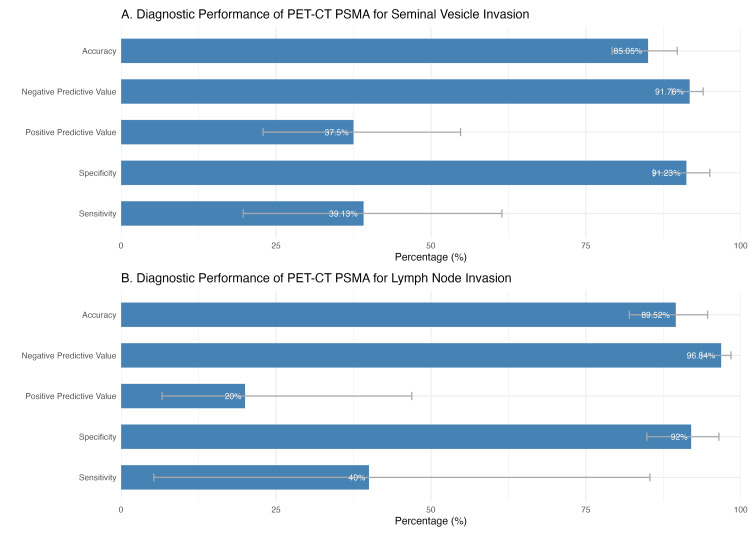
Diagnostic accuracy of PET-CT PSMA for seminal vesicle and lymph node invasion This figure illustrates the diagnostic performance of PET-CT PSMA in detecting (A) seminal vesicle invasion and (B) lymph node invasion in prostate cancer patients. The sensitivity, specificity, PPV, NPV, and accuracy are displayed with their respective 95% confidence intervals. PET-CT PSMA: positron emission tomography-computed tomography with prostate-specific membrane antigen; PPV: positive predictive value; NPV: negative predictive value

PET-CT PSMA showed high specificity for detecting seminal vesicle invasion (91.23%) and lymph node invasion (92%), with negative predictive values (NPVs) of 91.76% and 96.84%, respectively, suggesting good reliability in ruling out disease when the test is negative. However, sensitivity was low, particularly for seminal vesicle invasion (39.13%) and lymph node invasion (40%), indicating that PET-CT PSMA may fail to detect a substantial number of true-positive cases (Figure 3). 

The positive predictive value (PPV) for lymph node invasion was notably low (20%), reflecting a high rate of false positives, likely influenced by the low prevalence of nodal metastases in the cohort. Despite this, overall accuracy was high, reaching 85.05% for seminal vesicle invasion and 89.52% for lymph node invasion.

## Discussion

We found a significant correlation and agreement between PET-CT PSMA and the final histopathological report, for both seminal vesicle invasion and lymph node invasion, reinforcing the utility of PET-CT PSMA as an accurate tool for preoperative staging and robotic surgical planning. However, the observed correlation was low, and the agreement expected by chance (kappa) was poor. This highlights that despite PET-CT PSMA's diagnostic accuracy, its ability to predict histopathological findings remains limited when considering correlation and agreement metrics.

From a diagnostic performance perspective, PET-CT PSMA demonstrated high specificity for detecting seminal vesicle invasion (91.23%) and lymph node invasion (92%), with NPVs of 91.76% and 96.84%, respectively, reinforcing its reliability in ruling out disease. However, sensitivity remained low, particularly for seminal vesicle invasion (39.13%) and lymph node invasion (40%), indicating that PET-CT PSMA may fail to detect a significant proportion of true-positive cases. The PPV for lymph node invasion was particularly low (20%), suggesting a high rate of false positives, likely influenced by the low prevalence of nodal metastases in our cohort. Despite these limitations, overall accuracy was high, reaching 85.05% for seminal vesicle invasion and 89.52% for lymph node invasion, supporting PET-CT PSMA's role in preoperative assessment but also cautioning against over-reliance on its positive findings.

Our findings align with previous reports on the diagnostic performance of PET-CT PSMA. Zhang et al. evaluated PET-CT PSMA in patients with suspected prostate cancer before biopsy, reporting a PPV of 89.19% and an NPV of 85.71%, with an area under the curve (AUC) of 0.867 [8]. Similarly, Erdem et al. analyzed the diagnostic accuracy of PET-CT PSMA in intermediate- and high-risk prostate cancer patients before radical prostatectomy, reporting a sensitivity of 0.60 and specificity of 0.96 in a per-patient analysis, but without assessing correlation or agreement [11]. Luiting et al. conducted a systematic review on PET-CT PSMA for detecting lymph node metastases in primary prostate cancer, reporting specificity ranging from 80% to 100%, while sensitivity varied between 33.3% and 100% [12]. Our cohort demonstrated a low correlation for lymph node invasion (rho = 0.232), supporting the notion that although PET-CT PSMA is highly specific, its sensitivity remains suboptimal and cannot replace pelvic lymph node dissection for excluding metastases.

Our study further contrasts with the findings of Jiao et al., who established an SUVmax cutoff of 5.30 to differentiate clinically significant prostate cancer from benign prostatic conditions, achieving a sensitivity of 85.85% and specificity of 86.21% [10]. The correlation between SUVmax and tumor extension, particularly with lymph node and seminal vesicle invasion, was a key finding also observed in our analysis. However, unlike previous studies that primarily report diagnostic accuracy metrics, our study evaluates correlation and agreement between PET-CT PSMA and histopathology, emphasizing its limitations in confirming true-positive cases.

Study limitations

Despite these findings, this study has several limitations. Its retrospective, single-center design may limit generalizability to other populations and institutions. Additionally, the sample only included patients who underwent both PET-CT PSMA and robot-assisted radical prostatectomy, which may introduce selection bias. The variability in imaging interpretation among radiologists and histopathological assessments among pathologists may also impact the consistency of results.

## Conclusions

Our findings confirm a significant correlation and agreement between PET-CT PSMA and the final histopathological report in detecting seminal vesicle and lymph node invasion. However, correlation and agreement were low, reinforcing the notion that PET-CT PSMA should be used with caution for predicting histopathological outcomes, particularly for true-positive detection. The study highlights that PET-CT PSMA is highly specific but has limited sensitivity, suggesting that while it is a useful preoperative tool, it should not replace histopathological confirmation for staging purposes.
